# Association Between Measures Derived From Children’s Primary Exfoliated Teeth and Psychopathology Symptoms: Results From a Community-Based Study

**DOI:** 10.3389/fdmed.2022.803364

**Published:** 2022-03-28

**Authors:** Erin C. Dunn, Rebecca V. Mountain, Kathryn A. Davis, Ida Shaffer, Andrew D. A. C. Smith, Danielle S. Roubinov, Pamela Den Besten, Felicitas B. Bidlack, W. Thomas Boyce

**Affiliations:** 1 Psychiatric and Neurodevelopmental Genetics Unit, Center for Genomic Medicine, Massachusetts General Hospital, Boston, MA, United States,; 2 Department of Psychiatry, Harvard Medical School and the Massachusetts General Hospital, Boston, MA, United States,; 3 Henry and Allison McCance Center for Brain Health, Massachusetts General Hospital, Boston, MA, United States,; 4 Department of Oral and Craniofacial Sciences, University of California, San Francisco, San Francisco, CA, United States,; 5 Mathematics and Statistics Research Group, University of the West of England, Bristol, United Kingdom,; 6 Department of Psychiatry and Behavioral Sciences, University of California, San Francisco, San Francisco, CA, United States,; 7 Forsyth Institute, Cambridge, MA, United States,; 8 Department of Developmental Biology, Harvard School of Dental Medicine, Boston, MA, United States,; 9 Department of Pediatrics, University of California, San Francisco, San Francisco, CA, United States

**Keywords:** teeth, biomarkers, prevention, pediatric, internalizing symptoms, externalizing symptoms

Mental disorders are among the most disabling health conditions globally. However, there remains a lack of valid, reliable, noninvasive, and inexpensive biomarkers to identify (at an early age) people who are at the greatest risk of experiencing a future mental health condition. Exfoliated primary teeth, when used in combination with established and emerging tools (e.g., family history, imaging, genetics, epigenetics), may provide important additional insights about vulnerability to mental illness. Teeth are especially promising because they develop in parallel with the brain and maintain a permanent record of environmental insults occurring during prenatal and perinatal development. Despite their potential, few empirical studies have investigated features of exfoliated teeth in relation to mental health. Here, we used micro-CT imaging to test the hypothesis that measures derived from exfoliated primary incisors associated with psychopathology symptoms in a community-based sample of children (*n* = 37). We found that enamel volume (β = −0.77, 95% CI, −1.35 to −0.18, *P* = 0.01) had large negative associations with internalizing symptoms, and enamel mineral density (β = 0.77, 95% CI, 0.18–1.35, *P* = 0.01) had large positive associations with internalizing behavioral symptoms, even after stringent control for multiple testing. Pulp volume (β = −0.50, 95% CI, −0.90 to −0.09, *P* = 0.02) had a moderately-large negative association with externalizing behavioral symptoms, though these associations did not survive multiple testing correction. These results support the ongoing investigation of teeth as potential novel biomarkers of mental health risk.

## INTRODUCTION

Mental disorders constitute ~13% of the global burden of disease and are one of the most disabling groups of health conditions worldwide (1, 2). Mental disorders often begin early in life; in 50% of people with a mental disorder, the condition was diagnosed by early adolescence (3). In children, mental health problems are often classified into two symptom clusters (4): *internalizing symptoms*, meaning emotional and behavior problems that are inwardly-directed, such as depression or anxiety and *externalizing* symptoms, meaning emotional and behavior problems that are outwardly-directed, such as conduct problems or attention deficit hyperactivity difficulties (5, 6). High levels of internalizing and externalizing symptoms in childhood, in turn, have been found to predict a variety of poor outcomes in adulthood, including a 2- to 6-fold increase in risk for adult psychiatric diagnoses (7), as well as increased risk for academic failure (8), substance abuse problems (9), and even suicidality (10). Such poor outcomes may be attributable, in part, to the fact that people with early-onset psychopathology symptoms often delay seeking care, by as much as a decade, at which point symptoms have progressed and become more severe (11–13). Together, these findings emphasize the importance of identifying youth who may be at high future risk of psychopathology so they can be directed to early interventions, which have been shown to be effective in reducing psychopathology symptoms [see reviews by (14, 15)].

Recent advances in biomarker science are starting to identify new objective tools, such as neuroimaging, genetic, and epigenetic markers, which show promise in being able to one day help guide population-based risk stratification efforts and possibly even identify individual youth at-risk. Neuroimaging studies, for example, have identified associations between structural and functional markers (e.g., cortical thinning, decreased amygdala activity) and poor psychiatric outcomes in children (16–19). Genome-wide association studies (GWAS) have led to the creation of disease-specific and cross-disorder polygenic risk scores, which themselves have been associated in the population with up to about 5% of the variability in risk for psychopathology symptoms or neurodevelopmental phenotypes (20–22). Relative to self-reports of family history or children’s current psychopathology symptoms, biomarkers have a number of advantages. For instance, they are objective (rather than subjective) indicators and therefore might be more precise in characterizing population-level risk. Moreover, they could provide more precise information about underlying biological mechanisms shaping disease risk than broader self-report tools. Yet many biomarkers are currently limited due to their invasive nature and costly implementation on a population scale. New noninvasive and cost-effective biomarkers are therefore needed. With a broader suite of available tools, both researchers and clinicians would have more information (beyond family histories and symptom or diagnostic measures) to identify children who may benefit from early intervention.

Recently, we outlined a conceptual model—Teeth Encoding Experiences and Transforming Health (TEETH)—wherein we proposed that exfoliated primary teeth (also referred to as baby, deciduous, or milk teeth) could provide a new tool to guide primary prevention efforts in the fields of pediatrics as well as child and adolescent mental health (23). Our conceptual model was grounded in the observation that primary teeth develop on a parallel timeline with the brain and preserve a permanent record of prenatal and perinatal insults (23–25), which are potent risk factors for mental illness, per the prenatal programming hypothesis (26–28). Thus, we argued that inexpensive and noninvasive markers derived from exfoliated primary teeth—when used alongside existing tools—might serve as an additional early-warning sign for vulnerability to future psychopathology. If true, such insights could facilitate efforts to direct children who are vulnerable toward targeted interventions at the time when teeth are initially exfoliated, starting around age 6, which is years before the typical onset of many early life psychiatric disorders. Directing exfoliated teeth for such purposes therefore provides a possible alternative relative to throwing teeth away or storing them, as most parents often do (29).

By our review of the literature, several dozen studies have explored the properties of teeth as indicators of mental health risk, in a mixture of clinical, community-based, and population-based samples of youth (see Table 1). The overwhelming majority of research on teeth and mental health has investigated the associations of early life toxicants measured in primary teeth and risk for autism (30–36, 46) in particular, as well as internalizing and externalizing problems (40, 41), ADHD (37), schizophrenia (42, 43), and neurodegenerative disorders (38, 39) [see reviews by (47, 48)]. The most recent generation of these toxicant teeth studies, which date back to the early 2010s, use mass-spectrometry technology paired with laser ablation. This methodology, which requires sectioning the teeth to expose tooth structures, has identified time-specific windows in development when uptake of heavy metals (e.g., lead, manganese, etc.) differentiated cases with psychiatric disease from controls without disease. For example, in one of the largest and most robust studies, Mora and colleagues (40) found in a study of exfoliated primary teeth from 248 school-aged children that higher pre- and post-natal levels of magnesium in dentin were associated with worse internalizing and externalizing symptoms in young children, but improved cognitive abilities in young boys.

Outside of research on toxicants, a smaller body of research on teeth and mental health risk also exists, revealing connections between other aspects of primary teeth and psychopathology risk. For instance, adopting an approach focusing on the histology of the tooth (meaning, its structure) rather than its chemical composition, Kurek and colleagues (44) investigated how the presence of tooth growth lines, which are similar to the rings in a tree marking its age, associated with risk for autism in a sample of boys. The authors found that boys with autism were more likely than their peers without autism to have a greater number of a certain type of growth line, called accentuated growth marks or stress lines, which indicate some type of stressor occurred during enamel formation. Similarly, Boyce et al. (45) found markers of reactivity in the stress hormone cortisol associated with reduced average thickness and less mineralized enamel in a community-based sample of youth. Notably, sample sizes of studies examining toxicants or other features of teeth in relation to mental health studies are small by the standards of clinical or epidemiological studies (varying from n of 12 to 248); however, these sample sizes are considered typical in size for anthropological work. Though limited in total number, this collection of observational studies collectively suggest opportunities to bridge the fields of pediatric dentistry with child and adolescent psychiatry.

In the current study, we empirically tested the tooth-to-mental health risk part of our conceptual model (23) by analyzing data from a community-based study of kindergarten-aged youth. We used nondestructive micro-CT imaging to investigate the extent to which children’s exfoliated primary teeth associated with levels of child internalizing and externalizing symptoms. In this report, we provide the first evidence that basic morphological properties of children’s exfoliated primary teeth may serve as indicators of vulnerability to child psychopathology.

## METHODS

### Participants

We analyzed exfoliated primary mandibular central incisors from 37 kindergarten-aged children (20 boys; 17 girls; ranging in age from 5.9 to 6.8 years) enrolled in the Peers and Wellness Study (PAWS), a longitudinal school-based study of children attending public school in the California Bay Area (49, 50). The original PAWS study (*n* = 338) was designed to investigate the role of social dominance status, family environment, and biological responses to adversity on child physical and mental health. In 2004–2006, a dental tissue sub-study was launched, which recruited a subsample of PAWS children during their first grade year who had lost a tooth during the 9 months of the academic year, and donated at least one tooth to the study. All children donated incisors, which are typically the first primary teeth to exfoliate and begin forming during the second trimester of prenatal life and reach full maturation by the end of the first year of life. Incisors are comprised of three dental compartments: enamel (the white outermost part of the tooth), dentin (the layer below enamel), and pulp (the center part of a tooth consisting of connective tissue, blood vessels, and cells). Each child donated one or more incisors. However, only one tooth per child was included in this analysis (teeth were selected for analysis randomly). The teeth were free of dental cavities or developmental dental anomalies.

### Measures

#### Tooth Measures

Using micro-CT and Amira 6.5 analysis software (Thermo Fisher Scientific), we derived six measures from each incisor: (1) tooth size (micrometers; mm^2^), (2) enamel volume (mm^3^), (3) enamel density (Hounsfield units; HU), (4) dentin density (HU), (5) pulp volume (mm^3^), and (6) pulp shape (mm).

**FIGURE 1 |:**
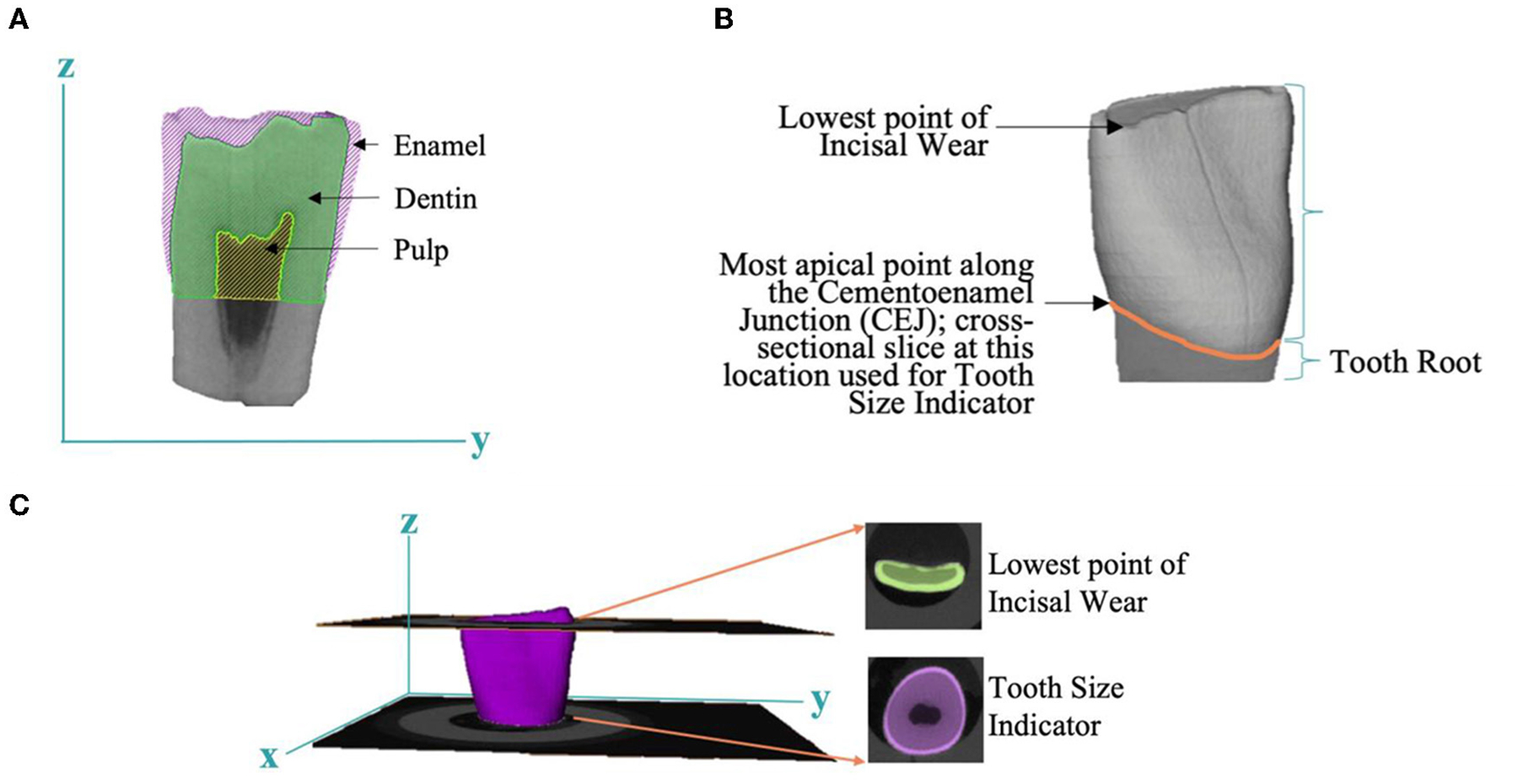
Tooth-based measures derived by micro-CT. **(A)** A 2D image highlighting the anatomy of the dental compartments: enamel, dentin, and pulp. **(B)** Estimation of tooth size based on most apical point along the cementoenamel junction (CEJ). **(C)** Evaluation of bruxing factor, or tooth wear, derived from lowest point of incisal wear and tooth size indicator.

After micro-CT scanning, images were uploaded into Amira and all teeth were spatially aligned against a reference tooth, using a Euclidean metric algorithm, allowing for consistent comparison between samples. A 3D Gaussian filter was applied to define the boundaries between the dental compartments (i.e., enamel, dentin, pulp). In the segmentation editor, the tooth root was cropped along the cementoenamel junction (CEJ) and analysis was performed on the tooth crown. Each dental compartment was assigned by relative density and compartment edges were refined with single voxel erosion and smoothing to clearly define each dental compartment (Figure 1A).

*Enamel and pulp volume* were measured by simple voxel counting within the enamel and pulp compartment, respectively. *Relative density of enamel and dentin* were assessed by averaging voxel intensity in each separate dental tissue. *Pulp shape* was calculated by a 3D sphere filling method which averages the diameters of the largest spheres that fit within the boundaries of the pulp chamber.

We observed loss of incisal tooth structure on 98% of the sampled teeth. This loss was likely associated with bruxing, or grinding, and is a common feature of pediatric dentition. The varying amounts of incisal wear did not allow for the direct use of total volume as the most accurate measure of tooth size. Therefore, we used the cross sectional area of the dentin and pulp at the most apical point of the cervical margin of the crown as a relative measure of *tooth size* (Figure 1B). Enamel, pulp volume, and pulp shape measures were normalized by *tooth size*. An index of bruxing (*bruxing factor*) was measured by taking the cross-sectional area of a slice through the lowest point of occlusal wear on each tooth and dividing this by *tooth size* (Figure 1C).

### Internalizing and Externalizing Symptom Measures

Psychopathology symptoms were assessed with the parent- and teacher-reported MacArthur Health and Behavior Questionnaire (51) and children’s self-report on the Berkeley Puppet Interview (52). Both of these measures are commonly-used, have excellent psychometric properties, and were designed specifically for children ages 4–8 years. Due to the benefits offered by multiple informants when evaluating children’s psychological symptoms, we used procedures outlined by Kraemer et al. (53) to conduct principal component analysis of potentially orthogonal reports from parents, teachers, and children and derive two multi-reporter indices of children’s internalizing and externalizing symptoms. The first component that was extracted reflected a trait dimension (individual differences in children’s internalizing or externalizing emotional and behavioral symptoms). This approach has been extensively used in prior studies (49, 54–56), including studies using both survey instruments (49).

### Covariates

We also analyzed data on the following social-demographic factors, which as described below were considered as possible covariates in our regression models: (1) child sex (coded as male vs. female), (2) child racial/ethnic background (coded as White vs. Other Race/Ethnicity); (3) family socioeconomic status; (4) children’s body mass index (kg/m^2^ evaluated at age 9), and (5) tooth wear (due to bruxing or chewing). Family socioeconomic status was measured as the standardized average (ranging from −3.13–1.64 in the total sample) of *parental education* and *family income* (57). Parental education was measured in 8 categories, from 1 = some grade school, to 8 = graduate or professional degree. Family income was measured in 15 categories (as a continuous variable), from 1 = <$10,000, to 15 = $400,000–499,999.

### Data Analysis

We first performed univariate analyses to understand the distribution of each study variable and then performed bivariate analyses to understand the pair-wise associations between each variable. We then evaluated associations between the six tooth-based measures and two psychopathology outcomes (internalizing and externalizing symptoms) using multiple regression performed in R 4.0.0. Multiple regression was preferable to univariate regression, because preliminary analysis confirmed the six tooth-based measures were reasonably correlated (see details below); thus, a given marker should be examined after controlling for the other markers. Covariates were selected for inclusion in regression models based on their having an association with at least one outcome, as we report below.

We included all tooth measures and selected covariates in each model; thus, each beta coefficient captures the estimated effect of that variable after controlling for the other teeth measures and covariates. To aid in interpretation and assist in the future reproducibility of our study results, we present results from both unstandardized and standardized regression models. Unstandardized regression models refer to analyses performed with the raw variables. Standardized regression models refer to analyses performed on standardized variables calculated by subtracting the variable mean and dividing by the standard deviation.

Partial R^2^ (meaning the amount of variability explained by the predictors above that explained by the covariates) are also presented and were adjusted for the number of predictors in model.

To ensure study rigor, we used 2-sided *P* (<0.05) to define statistical significance and implemented false discovery rate corrections (FDR) to adjust for multiple comparisons. We calculated variance inflation factors to assess whether our regression models were affected by multicollinearity.

## RESULTS

### Sample Descriptives

In the PAWS analytic sample of *n* = 37, 62.2% of children were White. The analytic sample also varied with respect to socioeconomic status (derived SES measure median = 0.4; SD: 0.8; min= −2.05; max = 1.1). Compared to children in the total PAWS sample, children in our analytic sample were more likely to be White (41% White in the total PAWS sample), and of higher SES (mean SES score = −0.01; SD: 0.88; min = −3.13; max = 1.64 in total PAWS sample) (57).

There was considerable variability in each of the tooth markers: (1) *tooth size* (mean = 9.42 mm^2^; SD = 1.33; min = 7.18; max = 13.39); (2) *enamel volume* (mean = 4.39 mm^3^; SD = 0.87; min = 2.42; max = 7.21); (3) *enamel density* (mean = 13,556 HU; SD=377.5; min = 12,704; max = 14,148); (4) *dentin density* (mean = 8,207 HU; SD = 192.9; min = 7,755; max = 8,577); (5) *pulp volume* (mean = 1.19 mm^3^; SD = 0.54; min = 0.28; max = 3.04); and (6) *pulp shape* (mean-0.66; SD = 0.26; min = 0.02; max = 0.98). Internalizing (mean = −0.21; SD = 0.86; min = −1.72; max = 1.61) and externalizing symptoms scores were also variable (mean = −0.17; SD = 0.73; min = −1.23; max = 1.22).

### Bivariate Associations Between All Study Variables

Table 2 presents the pair-wise correlation between the six tooth markers, the five possible socio-demographic covariates, and the two psychopathology outcome measures. As shown there, no tooth measure individually showed an association with either psychopathology measure. However, the significant correlations between the tooth measures mean it is inappropriate to consider univariate regression with single tooth measures. Hence, we proceeded to conduct multiple regression. Because socioeconomic status and racial/ethnic background were associated with internalizing symptoms, we included both of these variables as covariates in our multiple regression analyses.

### Multiple Regression Results

As shown in Table 3, several noteworthy findings emerged from our multiple regression analysis. First, enamel volume was negatively associated with internalizing symptoms, after adjusting for other predictors and sociodemographic covariates (standardized β = −0.77, 95% CI, −1.35 to −0.18, *P* = 0.01). The standardized regression coefficient suggested that internalizing symptoms decreased, on average, by 0.77 standard deviations per 1 standard deviation increase (equivalent to 0.87 mm^3^) in enamel volume, if all other predictors and covariates remain fixed.

Second, enamel density had a positive association with internalizing symptoms (standardized β = 0.77, 95% CI, 0.18–1.35, *P* = 0.01), suggesting internalizing symptoms increased, on average, by 0.77 standard deviations units per 1 standard deviation increase (equivalent to 378HU) in enamel density.

Of note, both of these standardized regression coefficients indicate a medium-sized effect (adjusted partial correlation = ± 0.42 for both, Cohens *f*^2^ = 0.21 for both). Moreover, both of these results were statistically significant, even after FDR correction for multiple testing.

Third, pulp volume had a negative association with externalizing symptoms (standardized β = −0.50, 95% CI, −0.90 to −0.09, *P* = 0.02) that would be considered a mediumsized effect (adjusted partial correlation = −0.39, Cohens *f*^2^ = 0.18). However, this association was not statistically significant after correction for multiple testing.

Collectively, these six tooth markers explained a further 15% and 20% of the variation in internalizing and externalizing symptoms, respectively, *above* that already explained by socioeconomic status and racial/ethnic background, two of the strongest predictors of child psychopathology (58). These findings were also detected after correcting for tooth size, thus were not explained by bruxing.

The largest variance inflation factor was 4.34, indicating multicollinearity was of minimal concern. We confirmed our regression models did not violate multiple regression assumptions, including the linearity assumption, by checking basic and partial scatterplots, and residual plots.

## DISCUSSION

The main conclusion of this study is that micro-CT-based measures from exfoliated primary teeth may provide new insights about child psychopathology symptoms, consistent with our TEETH conceptual model (23). Our results demonstrate for the first time that easily derived macro-level measures from children’s exfoliated primary teeth may provide new clues to help characterize children’s mental health risk. We showed that primary enamel volume was negatively associated with children’s internalizing symptoms, while enamel density was positively associated with internalizing symptoms. Furthermore, primary pulp volume was negatively associated with children’s externalizing symptoms.

Findings from the current study add to the existing literature by focusing on morphological properties in relation to child internalizing and externalizing symptoms. By our review of the literature, our study is the first of its kind. The current study is also an advancement of prior work, given our focus on investigating micro-CT-based macro-level measures of tooth features. Prior studies have primarily used other techniques to derive exposure data from teeth, including mass-spectrometry technology paired with laser ablation (33) or light microscopy (44). A major advantage of micro-CT measures is that they can be derived without destroying the tooth. Thus, micro-CT-based data has more potential to be incorporated into research, because it will allow for other future analyses of the collected teeth. Moreover, deriving data from micro-CT may be more cost-effective than other approaches. Although costs for lab procedures are variable and can change over time, depending on the research environment and its set-up (e.g., labs could have microCT covered as part of start-up; there are differences in core facility pricing based on internal versus external pricing for equipment time and the resolution of the scan), a “going rate” for the micro-CT we used is currently at about $100 per tooth.

What could explain our findings regarding the negative association between enamel volume and internalizing symptoms, and the positive association between enamel density and internalizing symptoms? We hypothesize these findings may reflect several biological processes in development. Primary tooth enamel is formed in stages, beginning during the second trimester of prenatal life and ending during the first year of postnatal life for incisors (59, 60). The full thickness of the enamel matrix is first laid down in the secretory stage, and then the matrix mineralizes to reach its full density in the maturation stage (61, 62). The rate of enamel matrix apposition is associated with the circadian rhythm (63); thus, the association between reduced enamel volume and internalizing symptoms suggests either an earlier end to the secretory stage or possibly circadian rhythm-related effects. The association between increased enamel density and internalizing symptoms indicates changes in either the onset and duration of maturation, or other mechanisms that affect how much mineral is formed and how much the enamel crystals grow during the maturation process. Because mandibular primary incisors are formed primarily in utero (64), these findings therefore reflect some type of aberrations occurring during prenatal development.

Should this work be replicated in future studies, these findings have potential important implications for both timely intervention and increasing our understanding of the etiology of psychopathology. For example, depressive and anxiety problems generally arise by 7 years of age in 1.1 and 8.5% of children, respectively (65). Children begin losing their first teeth by around age 6. Thus, if validated as a biomarker, primary teeth could potentially be used to identify high risk children, before the first signs of psychopathology symptoms are likely to emerge. These macro-level measures derived from microCT imaging of the exfoliated tooth may also one day be applicable to less precise but more accessible forms of tooth-based measurement, such as X-rays, which are routinely performed by dental professionals during pediatric primary care visits. Further, as primary teeth develop in an incremental manner during sensitive periods of development, they may preserve an important record of insults and their timing that could shed light on the causes of psychopathology symptoms (23).

There were several strengths of this study. For one, our measure of psychopathology symptoms came from a composite of scores from multiple reporters. Prior studies have shown multiple reporters often provide more information about child psychopathology symptoms than a single reporter alone (66). Second, we assess tooth morphology using non-destructive means. The possibility to acquire information about psychopathology risk from intact teeth might enable more parents to donate teeth to science (because the tooth could be returned to the parent, if desired, to hold as a keepsake). Moreover, intact teeth can also be further interrogated for other analyses in the future, though radiation through microCT must be noted. Third, our study was able to correct for tooth size, ensuring that our results were not explained by between-child differences in the size of dentition. Finally, we were rigorous in our analysis: we controlled for potential covariates, tested for regression model assumptions, evaluated the possibility of multicollinearity, and adjusted for multiple testing.

This study had several limitations. First, children in this study were identified in first grade, thus we lacked information about children’s prenatal and perinatal life. If available, such data could have enabled us to evaluate the extent to which tooth abnormalities reflected the biological imprints of maternal pregnancy (e.g., social support; maternal history of psychopathology; stressors) or child factors during prenatal or early development (e.g., gestational age; exposure to medications or exposure to toxicants) (67). Indeed, we recently showed in a population-based sample of 70 youth that maternal depression and perceptions of social support were associated with the thickness of the neonatal line, a prominent growth marker in exfoliated teeth thought to be indicative of stressful birth conditions (67). Caution is therefore warranted in interpreting these results, as our findings would be biased away from the null if early life factors shaped both tooth formation and child psychopathology. Second, we did not have information on the potential biological mechanisms linking tooth measures with child psychiatric symptoms. Although establishing an initial association between two factors is a key first step, future studies are needed to both replicate these associations and further unravel what biological mechanisms in development could be explaining those associations. Third, our study was limited in sample size, but comparable to prior work exploring the relationship between tooth-markers and mental health (42). Our study was also comprised of mostly European-ancestry children and thus was not representative of more racially/ethnically diverse groups of youth, limiting the generalizability of these findings. Finally, data were originally collected more than two decades ago from a sample of children who were not followed longitudinally to adolescence. Funding for the study concluded and data collection did not continue beyond first grade. Thus, we lack any information on specific psychiatric diagnoses children may have developed later in life. However, early in life, children often move dynamically in and out of diagnostic classifications of mental disorders (68) and intermediate behavioral risk factors are important and robust predictors of psychopathology in adulthood. In fact, a recent systematic review concluded that elevated symptomatology in childhood was more strongly associated with adult mental health disorders than childhood diagnoses (69).

Exfoliated primary teeth hold great appeal as potential biomarkers. Exfoliated primary teeth can be collected easily, as nearly every child globally exfoliates primary teeth. Exfoliated teeth can also provide nuanced data on the occurrence and timing of otherwise hard to measure retrospective events. The early age of natural tooth shedding also means insights gained from exfoliated teeth could help guide risk-stratification and/or prevention efforts for youth at-risk (23). However, as we have reported (29), teeth are different from other biospecimens: they are finite; parents view tooth exfoliation as a developmental milestone bearing nostalgic value, and parents practice a variety of rituals with their children once their teeth are exfoliated. These and other factors may determine if parents donate their children’s teeth to science, and if so which and how many teeth they donate.

As with any emerging biomarker, high standards for scientific rigor are needed when conducting and reporting research on teeth. Such scientific rigor should exist across all stages of the research process, including unbiased study designs, data analysis, as well as the interpretation and reporting of results. Through such rigor, we can help ensure findings are reproducible and in what populations and contexts such findings are validated. Further, as research on primary teeth also progresses, researchers can incorporate the valuable lessons learned from other biomarkers to avoid the pitfalls that prior work encountered, including the importance of extending studies to more diverse samples, and help determine the extent to which markers from teeth provide distinct or similar information to other measures.

We hope the results of the current study will encourage researchers to test our TEETH conceptual model more fully and characterize potential links in this “tooth-brain-axis,” including the impact of early life stress on tooth formation. Replication and validation of these findings are needed, which can help determine the usefulness of teeth as indicators of mental health risk. Through such work, teeth could one day be used as an additional tool—alongside information on family history and other biomarkers—to identify children at high-risk for future mental disorders.

## Figures and Tables

**TABLE 1 |:** Examples of empirical research reporting associations between properties of teeth and mental health outcomes.

Analysis method	Mental health outcome	Description	References
Early-life toxicant exposure (laser ablation)	Autism	Studies typically report differences in prenatal and early postnatal levels of heavy metals, as well as in the cyclic variation of metal concentrations, between children diagnosed with autism and controls.	(30–36)
	ADHD	Regularity and complexity of elemental cycles (for cobalt, lead, and vanadium) was reduced in children diagnosed with ADHD compared to those diagnosed with autism, along with other element specific features.	(37)
	ALS	Metal levels are higher in ALS cases than controls during specific windows of development in childhood and adolescence.	(38, 39)
	Internalizing and Externalizing Symptoms	Manganese exposure during specific developmental windows was associated with poorer behavioral outcomes in children.	(40, 41)
	Schizophrenia	Higher exposures to metals (e.g., lead, lithium) early in life were found in individuals with diagnoses of schizophrenia relative to controls.	(42, 43)
Growth Lines (histology)	Autism	An increased number of accentuated lines in tooth enamel were more common in children with an autism spectrum disorder diagnosis than controls.	(44)
Morphology (microCT)	Cortisol Reactivity	Thinner and lower density tooth enamel was associated with higher cortisol reactivity among school-aged children.	(45)

ADHD, attention deficit hyperactivity disorder; ALS, amyotrophic lateral sclerosis.

**TABLE 2 |:** Correlations between the six tooth measures, the five possible covariates, and the two outcomes (*n* = 37).

	1.	2.	3.	4.	5.	6.	7.	8.	9.	10.	11.	12.
1. Tooth size (mm^2^)												
2. Enamel volume (mm^3^)	0.79***											
3. Enamel density (HU)	0.34*	0.46**										
4. Dentin density (HU)	0.11	0.14	0.78**									
5. Pulp volume (mm^3^)	0.30	0.42**	−0.03	−0.13								
6. Pulp shape (mm)	−0.37*	−0.24	−0.21	−0.10	−0.41*							
7. Child sex (female = 1)	0.12	0.00	0.01	−0.09	−0.12	0.00						
8. Child race (white = 1)	−0.09	−0.09	−0.07	−0.02	−0.26	0.00	0.29					
9. Family SES (continuous)	−0.22	−0.20	−0.23	−0.09	−0.25	0.16	0.16	0.55**				
10. Child BMI (continuous)	0.15	0.01	−0.05	−0.11	0.01	0.11	0.14	−0.31	0.02			
11. Bruxing factor tooth	−0.23	−0.03	−0.03	−0.12	−0.19	0.13	0.14	−0.14	−0.11	−0.10		
12. Internalizing symptoms	0.07	−0.09	0.27	0.11	0.03	−0.15	0.06	−0.41*	−0.47**	0.29	0.15	
13. Externalizing symptoms	0.02	−0.19	0.05	−0.08	−0.39	−0.11	0.13	0.02	−0.20	0.13	0.12	0.63***

The six tooth measures are presented first, followed by the five possible covariates, and the two outcome measures. Pearson correlation values are presented for pair-wise correlations between all measures. Correlations with the binary variables (Child Sex and Child Race) can be interpreted as effect size correlations. Significance tests for Pearson correlations were calculated in the usual way. Significance tests for association between binary and continuous measures were calculated using t-tests. Significance tests for association between the two binary variables were calculated using Fisher’s exact test.

* p < 0.05,

** p < 0.01,

*** p < 0.001.

**TABLE 3 |:** Results of multiple linear regression analyses examining the association between tooth measures and levels of internalizing and externalizing symptoms, adjusted for socioeconomic status, race/ethnicity, and other tooth-based measures (*n* = 37).

	Internalizing symptoms			Externalizing symptoms			VIF
Predictors	Unstandardized Beta (95% CI)	Standardized β (95% CI)	*P* value	Unstandardized Beta (95% CI)	Standardized β (95% CI)	*P* value	
Tooth size (mm^2^)	0.22 (−0.10, 0.55)	0.35 (−0.15, 0.85)	0.17	0.14 (−0.15, 0.43)	0.25 (−0.28, 0.79)	0.34	3.18
Enamel volume (mm^3^)	**−0.76** (**−1.35,−0.18)**	**−0.77 (−1.35, −0.18)**	**0.01** *	−0.32 (−0.85, 0.20)	−0.38 (−1.00, 0.24)	0.22	4.34
Enamel density (HU)	**0.002 (0.0004, 0.003)**	**0.77 (0.18, 1.35)**	**0.01** *	0.0007 (−0.0005, 0.002)	0.35 (−0.27, 0.97)	0.26	4.33
Dentin density (HU)	−0.002 (−0.004, 0.0002)	−0.44 (−0.93, 0.05)	0.08	−0.002 (−0.004, 0.0003)	−0.43 (−0.96, 0.09)	0.10	3.07
Pulp volume (mm^3^)	0.14 (−0.47, 0.75)	0.09 (−0.29, 0.47)	0.64	**−0.67 (−1.22, −0.12)**	**−0.50 (−0.90, 0.09)**	**0.02**	1.88
Pulp shape (mm)	−0.01 (−1.19, 1.16)	−0.004 (−0.35, 0.34)	0.98	−0.64 (−1.70, 0.42)	−0.36 (−0.60, 0.15)	0.75	1.55
**Partial R**^**2**^ **of predictors**		0.15			0.23		

HU, Hounsfield units; VIF, variance inflation factor.

We included all tooth measures and selected covariates in each model; thus, each beta coefficient captures the estimated effect of that variable after controlling for the other teeth measures and covariates. Covariates were selected for inclusion in models based on their having an association with at least one outcome. Both models included socioeconomic status and racial/ethnic background as covariates. To aid in interpretation and reproducibility of results, we present results from both unstandardized and standardized regression models. Unstandardized regression models refer to analyses performed with the raw variables. Standardized regression models refer to analyses performed on standardized variables calculated by subtracting the variable mean and dividing by the standard deviation.

Partial R^2^ (meaning the amount of variability explained by the predictors above that explained by the covariates) were adjusted for the number of predictors in model.

Bolded results are statistically significant at P < 0.05.

* Result remained statistically significant after FDR correction for multiple comparisons.

## Data Availability

The raw data supporting the conclusions of this article will be made available by the authors, without undue reservation.
